# Microleakage Evaluation of Temporary Restorations Used in Endodontic Treatment—An Ex Vivo Study

**DOI:** 10.3390/jfb14050264

**Published:** 2023-05-09

**Authors:** Siri Paulo, Ana Margarida Abrantes, Mariana Xavier, Ana Filipa Brito, Ricardo Teixo, Ana Sofia Coelho, Anabela Paula, Eunice Carrilho, Maria Filomena Botelho, Carlos Miguel Marto, Manuel Marques Ferreira

**Affiliations:** 1 Institute of Endodontics, Faculty of Medicine, University of Coimbra, 3000-548 Coimbra, Portugal; 2 Coimbra Institute for Clinical and Biomedical Research (iCBR), Area of Environment Genetics and Oncobiology (CIMAGO), Faculty of Medicine, University of Coimbra, 3000-548 Coimbra, Portugal; 3 Center for Innovative Biomedicine and Biotechnology (CIBB), University of Coimbra, 3000-548 Coimbra, Portugal; 4 Clinical Academic Center of Coimbra (CACC), 3004-561 Coimbra, Portugal; 5 Institute of Biophysics, Faculty of Medicine, University of Coimbra, 3000-548 Coimbra, Portugal; 6 Institute of Integrated Clinical Practice, Faculty of Medicine, University of Coimbra, 3000-075 Coimbra, Portugal; 7 Institute of Experimental Pathology, Faculty of Medicine, University of Coimbra, 3000-548 Coimbra, Portugal

**Keywords:** endodontic treatment, sealing ability, microleakage, temporary material, nuclear medicine

## Abstract

(1) Background: Coronal microleakage can lead to endodontic treatment failure. This study aimed to compare the sealing ability of different temporary restorative materials used during endodontic treatment. (2) Methods: Eighty sheep incisors were collected, uniformized in length, and access cavities were performed, except for in the negative control group, where the teeth were left intact. The teeth were divided into six different groups. In the positive control group, the access cavity was made and left empty. In the experimental groups, access cavities were restored with three different temporary materials (IRM^®^, Ketac™ Silver, and Cavit™) and with a definitive restorative material (Filtek Supreme™). The teeth were submitted to thermocycling, and two and four weeks later, they were infiltrated with ^99m^TcNaO_4_, and nuclear medicine imaging was performed. (3) Results: Filtek Supreme™ obtained the lowest infiltration values. Regarding the temporary materials, at two weeks, Ketac™ Silver presented the lowest infiltration, followed by IRM^®^, whereas Cavit™ presented the highest infiltration. At four weeks, Ketac™ Silver remained with the lowest values, whereas Cavit™ decreased the infiltration, comparable to IRM^®^. (4) Conclusion: Regarding temporary materials, Ketac™ Silver had the lowest infiltration at 2 and 4 weeks, whereas the highest infiltration was found in the Cavit™ group at two weeks and in the IRM^®^ group at 4 weeks.

## 1. Introduction

Endodontic treatment is based on chemical and mechanical debridement, root canal filling, and later definitive crown restoration, to eliminate bacteria and prevent reinfection [1,2,3,4]. Before the treatment, it is fundamental to remove dental caries and infiltrated old restorations, or perform pre-endodontic restorations, to avoid microleakage, which is defined as the diffusion of saliva, microorganisms and their products, ions, and molecules to the canals, which can lead to treatment failure [3,5,6,7,8]. Several reports identified bacteria strains from the oral cavity, such as *Prevotella intermedia, Porphyromonas gingivalis, Enterococcus faecalis, Streptococcus viridans*, *Staphylococcus* sp., and *Enterococcus faecalis*, as responsible for persistent infections and endodontic failures after endodontic treatment [1,7,9].

To avoid reinfection during or after the treatment and consequently improve prognosis, a proper provisional restoration must be performed during the endodontic treatment [1]. Even after the root canal filling, until the final restoration is performed, temporary fillings are still essential since the obturated root canal system, exposed to saliva, is also susceptible to microleakage [10,11]. An infiltration of 79–85% of the obturated root canal system in a 3- to 56-day interval [12], and a microleakage of *S. epidermidis* in 19 days and of *P. vulgaris* in 42 days in teeth without an efficient restoration [10,11] was reported, supporting the importance of temporary restorations for treatment success. 

The ideal temporary material should avoid contact between the root canal system and the oral environment and be resilient to abrasion and compression. It should display low porosity, dimensional stability, good sealing, and reasonable aesthetics, and have the capacity to prevent the canal system from becoming contaminated by saliva, fluids, and microorganisms [3,5,6,13]. In addition, to be an effective barrier material, a minimum of 4 mm thickness is necessary [1,5,14,15,16]. Nowadays, the temporary materials most used in clinical practice are Cavit™ (3M ESPE, Seefeld, Germany), a calcium sulfate-based cement; Ketac Silver™ (3M ESPE, Seefeld, Germany), a glass ionomer cement with silver particles; and IRM^®^, a reinforced zinc-oxide-eugenol cement (Dentsply, Milford, DE, USA). The choice of the material to be used depends on the clinical needs of each case, such as duration of use, dimensional stability, abrasion resistance, stabilization of intracanal medication, and adaptation to more complex access cavity formats [3,17]. Several studies, mostly in vitro, have evaluated the seal capacity of these materials, and there is evidence that microleakage occurs to different degrees for most temporary materials available, and none of them can entirely prevent microleakage from the 1st to the 14th day [1,2,3,4,18].

Several studies have been performed on this topic, but the obtained data presents contradictory results [1,3,4,19,20,21,22,23]. This may be due to different testing materials, evaluated time points, and experimental protocols [4,14,20,21,22,23,24,25]. However, the main reason is the use of different methodologies to appraise microleakage, namely dyes, radioisotopes, bacteria, or their sub-products [1,26,27]. Most studies use dyes such as methylene blue, but more sensitive methods, such as nuclear medicine, can provide more accurate results [26,27].

Considering its influence on the treatment’s success, evaluating which materials can successfully prevent microleakage is fundamental. Therefore, the three most used temporary restoration materials and a definitive restoration material were compared in the present study. The null hypothesis was that there were no differences in microleakage between the three temporary restorative materials—Cavit™, Ketac Silver™, and IRM^®^ at 2 and 4 weeks. A second null hypothesis was that there were no differences in microleakage between the temporary materials and the definitive restorative material—composite resin Filtek Supreme™. 

## 2. Materials and Methods

### 2.1. Sample Preparation

For this study, sheep teeth were used. The teeth were obtained post mortem at a food sector abattoir. The animals were handled and euthanized according to the Portuguese (DL 98/96, Art. 1°) and European Legislation concerning animal welfare (EFSA, AHAW/04-027). Eighty incisor teeth from two-year-old sheep were collected and cleaned by removing soft tissues and other residues. The teeth were then disinfected in azide chloride solution (0.01 g/mL) for three days [27]. 

**Figure 1:**
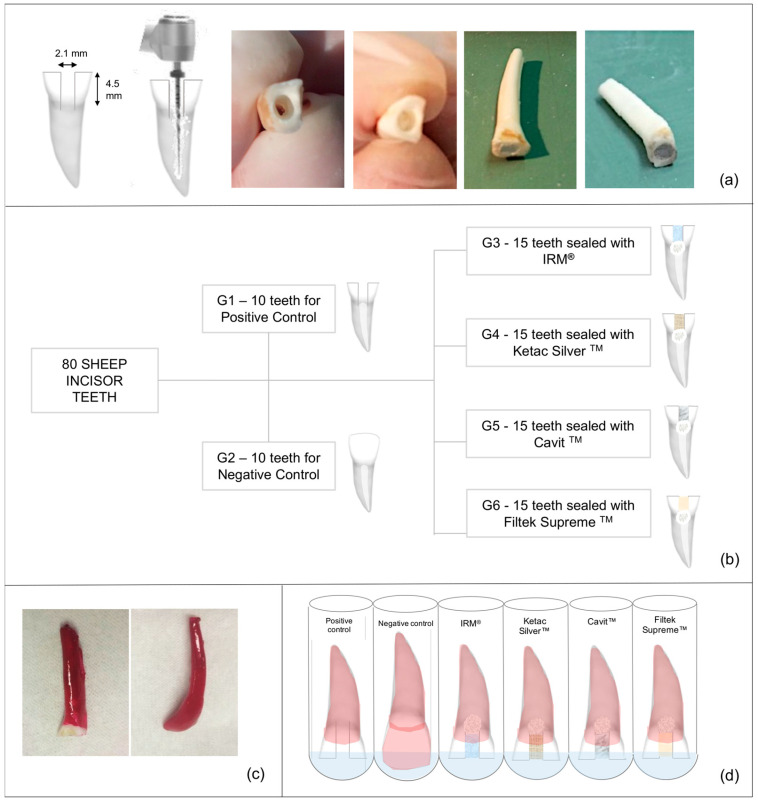
Study flowchart. (**a**). Preparation of uniformized samples—teeth were sectioned 2 mm above the cementoenamel junction; an access cavity was made with a round bur; teeth were instrumented and had Teflon condensed at the bottom of the cavities, leaving a 4 mm height to restoration placement. (**b**). Groups distribution. (**c**). Preparation for nuclear medicine analysis—tooth impermeabilized with two layers of nail polish, in all root surface besides in the last 1 mm of the access cavities (except in the negative group in which all the tooth surface was covered). (**d**). Teeth in test tubes, immersed in ^99m^TcNaO_4_.

To obtain uniformized samples of 16 mm in length, the teeth were sectioned 2 mm above the cementoenamel junction, except in the negative control group. After this, [4,6,20,27] a spherical diamond turbine drill with a 2.1 mm diameter at high speed and constant irrigation was used to create access cavities of 2.1 mm (length) × 2.1 mm (width) × 4–4.5 mm (height) [18], measured with a periodontal probe, to allow the minimum thickness of 4 mm for the restoration material (Figure 1a) [1,5,14,15,16]. The remaining pulp tissue was removed using an endodontic K30 file with sodium hypochlorite (2.5%) irrigation. Next, a glide path with manual files K10 and K15 was achieved, followed by canal instrumentation using the ProTaper NEXT™ (PTN) system (Dentsply Sirona), triggered by X-SMART™ (Dentsply Sirona) with a constant rotation of 300 RPM and a torque of 3. A sequence of the PTN^TM^ files PG, X1, X2, X3, and X4 or X5 was used, depending on the diameter of the different canals (Figure 1a). Next, irrigation with sodium hypochlorite (2.5%) and permeabilization with a manual K10 file were performed between each file. Following this, the canals were irrigated with EDTA (15%) and then with 3 mL of 0.9% saline solution for 3 min to neutralize EDTA. Finally, all the canals were dried with paper points.

Finally, the teeth were randomly divided into two groups of 10 for the positive and negative control groups and four experimental groups of 15 teeth each (Figure 1b). Then, Teflon was placed in the access cavities and condensed at the cavities’ bottom, at the canal’s entrance, to support the temporary material [28].

### 2.2. Temporary Restoration Material Fillings

Except for Group 1 (teeth with empty access cavity—positive control) and for Group 2 (intact teeth—negative control), the remaining access cavities were filled with different temporary restorative materials (Table 1). 

The materials were prepared and used following the manufacturer’s instructions. For IRM^®^, a 1:1 mixture was prepared until a stable consistency was attained. The polymerization occurred after a few seconds. Ketac Silver™ was activated with vibration for 10 s. The material was then applied with a Ketac Aplicap™ Applicator (3M ESPE, Seefeld, Germany). This is a self-cure material with a short working time, and its setting starts within seconds. Cavit™ is a pre-mixed material that does not require any preparation. It was taken directly from the container, applied to the tooth, and left to set for 4 h. Before the restoration with Filtek Supreme™, a selective enamel etching with orthophosphoric acid (37%) was performed, and Scotchbond™ Universal dental adhesive (3M ESPE, Seefeld, Germany) was applied, according to the manufacturer’s instructions. The composite was inserted in the cavities in 2 mm layers. The adhesive system and each layer of the composite resin was photopolymerized.

The same experienced operator performed all the temporary restorations to avoid technical bias. Upon the crown sealing, all teeth were placed in 0.9% saline solution at room temperature to mimic the clinical environment [14,23,25,27].

### 2.3. Thermocycling

The teeth were subjected to thermic stress to simulate the aging of the restoration materials that occurs clinically. For this step, teeth were alternately placed in baths at 5 ± 5 °C and 55 ± 5 °C for 30 s periods in each bath, totaling 500 cycles. These temperatures, intervals, and the number of cycles were chosen according to the ISO/TS 11405: 2015 recommendations. The 30 s interval mimics the latency time of the oral environment to recover its normal temperature after exposure to hot or cold food or drinks [29,30,31]. The thermocycling set-up was chosen considering 10,000 cycles/year occurs. After thermocycling, the samples were kept in saline until nuclear medicine analysis.

### 2.4. Nuclear Medicine

Two and four weeks after thermocycling, all samples were dried, sealed apically with cyan acrylate [3] and impermeabilized with 2 layers of nail polish (Catrice Cosmetics) in all root surface besides in the last 1 mm of the access cavities, except in the negative group in which all the tooth surface was covered [3,15] as depicted in Figure 1c.

Impermeabilized teeth were placed in a sodium pertechnetate solution, ^99m^TcNaO_4_ (8 mCi/mL), immersing only the non-impermeabilized segment for 3 h (Figure 1d) [27]. Teeth were then washed in tap water at constant flow for 30 s each. After this, the teeth were dried with absorbent paper, and the nail polish was removed with a scalpel. 

Next, for each sample, a 512 × 512 pixel image was acquired in a gamma chamber (Millennium, New York, NY, USA) for 2 min. In each image, a region of interest (ROI) with the same pixel size was applied to obtain the total, maximum, and average values of micro infiltration. After the two-week analysis, the samples were again stored in the saline solution until the four-week evaluation [14,23,25,27]. At four weeks, the described nuclear medicine analysis was repeated. 

### 2.5. Statistical Analysis

The sample size of 15 specimens in experimental groups and 10 specimens in control groups was chosen in accordance with previously published similar studies [4,6,21,32]. The sample size was validated by a post hoc analysis of sample size calculation, which retrieved a confidence interval of 99.15%, and a size effect of 2.4, using G* Power 3.1 [33]. 

Statistical analysis was performed using IBM SPSS Statistics 28 software (IBM Corporation, Armonk, NY, USA) and data were presented as mean ± standard deviation (SD). The Shapiro–Wilk test was used to evaluate the normality of each population distribution, and the Levene test was used to assess the variance homogeneity. Parametric tests (ANOVA) were employed when normal distribution was observed, and non-parametric tests (Kruskal–Wallis) were used for non-normal distributions to assess multiple comparisons of the different temporary materials studied at the same time of assessment, with post hoc correction by Tukey’s test. To compare each of the different temporary materials at different evaluation times, the parametric Student’s *t*-test and the non-parametric Wilcoxon test were used according to sample normality. A family-wise 95% confidence level (*p* < 0.05) was applied to all statistical analyses. 

## 3. Results

**Figure 2:**
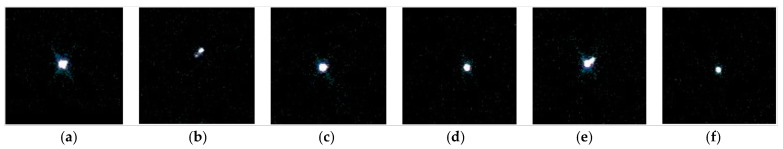
Representative scintigraphy images of positive control (**a**), negative control (**b**), IRM^®^ (**c**), Ketac^TM^ (**d**), Cavit^TM^ (**e**), and Filtek^TM^ (**f**) groups with uptake of ^99m^TcNaO_4_.

**Figure 3:**
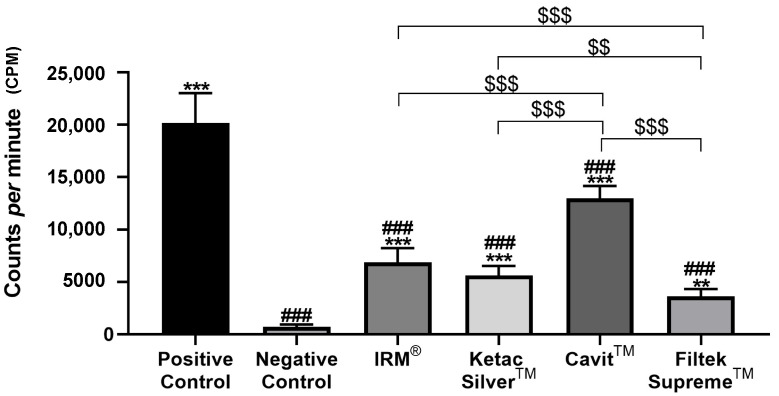
Total counts per minute (cpm) were obtained after infiltration with ^99m^TcNaO_4_, 2 weeks after restoration. ### means *p* < 0.001 (relative to the positive control); ** means *p* < 0.01 and *** means *p* < 0.001 (relative to the negative control); $$ means *p* < 0.01 and $$$ means *p* < 0.001 (comparisons between materials). Data are presented as mean ± standard deviation (SD).

**Figure 4:**
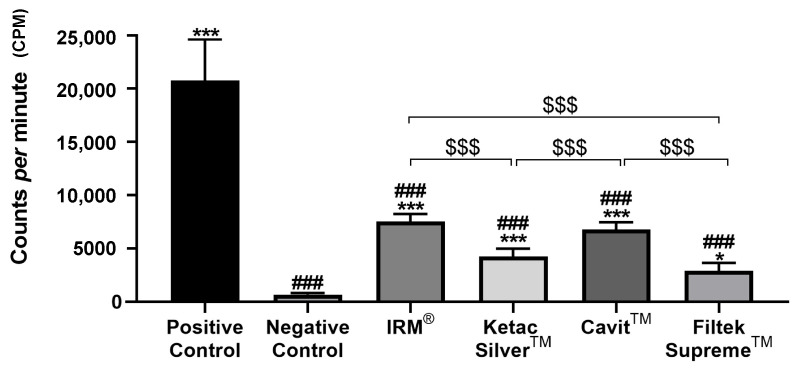
Total counts per minute (cpm) were obtained after infiltration with ^99m^TcNaO_4_, 4 weeks after restoration. ### means *p* < 0.001 (relative to the positive control); * means *p* < 0.05, and *** means *p* < 0.001 (relative to the negative control); $$$ means *p* < 0.001 (comparisons between materials). Data are presented as mean ± SD.

At 2 and 4 weeks, the positive group, i.e., teeth with cavity access performed but without restoration, displayed the highest counts per minute (cpm), indicating the highest microleakage across the different groups (Figure 2, Figure 3 and Figure 4, Table 2).

Conversely, the negative control group, composed of intact teeth without restoration, displayed the lowest levels of infiltration at the two time points (Figure 3 and Figure 4, Table 2). At both times, all tested materials presented a significantly lower infiltration than the positive control group and a higher infiltration relative to the negative control group (Figure 3 and Figure 4, Table 2).

At 2 weeks after restoration, Cavit™ displayed the highest values of ^99m^TcNaO_4_ infiltration, significantly higher than the other three tested materials, followed by IRM^®.^ (Table 2, Figure 3). 

At 4 weeks, Cavit™ and IRM^®^ showed similar infiltration, higher than that for Ketac™ Silver, which showed the lowest infiltration observed in the teeth with temporary restoration, identical to that observed at 2 weeks (Figure 4).

Comparing the infiltration between 2 and 4 weeks within each group, a significant reduction in counts per minute was found from 2 to 4 weeks in Ketac Silver^TM^ and with Cavit^TM^ groups, but not in Filtek Supreme^TM^ or with IRM^®^. No changes in infiltration from 2 to 4 weeks were observed in both positive and negative control groups (Table 2).

## 4. Discussion

The present study aimed to compare the sealing ability of three widely used temporary restorative materials. Temporary restorations are fundamental during and after endodontic treatment to prevent microleakage and ensure treatment success. Conversely, the lack of satisfactory temporary restorations can lead to bacteria infiltration, flare-up reactions, antibiotics administration, and, ultimately, a poor prognosis [1,17].

Regarding the experimental model chosen, using ex vivo models in dentistry is widespread and helpful [34]. Therefore, sheep teeth were selected since they show anatomic and histological similarities with human teeth and are commonly used in endodontic research [34,35,36]. In addition, they allow the necessary sample to be obtained in a reasonable time, which is a disadvantage of using human teeth, especially specific teeth such as incisors. 

During the experimental protocol, thermocycling was chosen to simulate the oral conditions, namely hot and cold food exposure [30]. In the oral cavity, temperature changes affect the marginal seal of restoration materials because the linear coefficients of thermal expansion of the materials and dental tissues are different [22,37]. This way, the temperature changes altered the linear coefficient of thermal expansion of both the tooth and the restorative material, mimicking oral conditions. In addition, the artificial aging induced by thermocycling includes an initial phase with hot water, accelerating the hydrolysis of unprotected collagen [38]. Consequently, the high coefficient of thermal expansion/contraction of the material generates stress in the tooth-restoration interface and creates spaces that favor the pathway to fluids and microorganisms [31]. Additionally, the teeth were stored in a saline solution until being evaluated. As suggested by ISO/TS 11405: 2015, storage in an aqueous medium allows the differentiating of materials that resist wet environments from those that do not. Since all materials are used in the oral cavity, which is a moist environment, the ability to resist wet environments is essential [21]. In addition, the two time points chosen simulate a period between appointments of 15 days, similar to clinical conditions. 

The obtained results show all tested materials presented microleakage, although to different degrees. The first null hypothesis was rejected since, at 2 weeks, Cavit™ presented a significantly higher microleakage when compared with IRM^®^ and Ketac™ Silver. At 4 weeks, Ketac™ Silver presented significantly lower microleakage compared with IRM^®^ and with Cavit™. The second null hypothesis was also rejected since composite resin Filtek Supreme™ presented significantly lower microleakage at 2 weeks compared with the three temporary restorative materials, and at 4 weeks, when compared with IRM^®^ and with Cavit™.

Overall, Cavit™ presented significantly high levels of infiltration at 2 weeks, which decreased abruptly at 4 weeks, whereas IRM^®^ microinfiltration remained stable from 2 to 4 weeks. Ketac™ Silver presented significantly lower infiltration at 2 and 4 weeks. 

Cavit™ is a pre-mixed auto-polymerized material that contains zinc oxide and synthetic resins without eugenol. Cavit™ is prefabricated in three different forms: Cavit™ (pink), Cavit™-W (white), and Cavit™-G (grey). Cavit™ and Cavit™-W have different concentrations of zinc sulfate and zinc oxide, resulting in a hardness increase for Cavit™ and an adhesion increase for Cavit™-W [2]. In this study, Cavit™ was used, which has an indication to be used during endodontic treatment. Cavit™ is endowed with favorable properties for crown sealing, such as hygroscopic expansion due to water absorption [2]. However, it is also characterized by a low mechanical resistance and slow setting [2,15]. Other studies evaluating this material present contradictory results, probably related to experimental set-up and evaluation periods [3,4,22,23,39,40]. In the present study, Cavit™ presented an abrupt decrease in infiltration values from 2 to 4 weeks. The slow setting may justify the high infiltration at 2 weeks. At 4 weeks, the hygroscopic properties and the elevated coefficient of linear expansion resulting from water absorption (approximately double that of IRM^TM^) seem to contribute to the sealing improvement and infiltration decrease. Nevertheless, microleakage was superior to other materials at both time points, and thus it is not an appropriate material for longer periods. If a longer time period were to be evaluated, infiltration would be expected to increase again since the existence of a high number of pores facilitates water absorption and adhesion degradation over time [22]. 

IRM^®^ is an auto-polymerized material that contains zinc oxide and eugenol. Although it is characterized by more difficult manipulation [15], it showed intermediate levels of infiltration. As for Cavit^TM^, several studies present non-consensual results for IRM^®^ infiltration. Similar to our results, some show IRM^®^’s sealing ability to be superior to that of Cavit™ at shorter evaluation times, whereas others present IRM^®^’s sealing ability to be inferior to that of Cavit^TM^ [4,18]. Again, different experimental setups and evaluating methodologies can explain the contradictory results. In the present study, IRM^®^’s infiltration values slightly increased from 2 to 4 weeks, without statistical difference. That can be related to the continuous release of eugenol, which is hydro-soluble and favors the detachment of particles from IRM^®^, increasing porosity [41]. In addition, the higher level of infiltration, when compared with that of Ketac^TM^, can be related to a variation of volume arising from polymerization contraction or to the non-homogeneous handmade mixture subjected to operator variability [23,42]. Nevertheless, this material demonstrated stability over the evaluation periods. 

Ketac™ is a metal-reinforced glass ionomer material with fine particles of silver fused with the glass. It has an ion-leachable alumino-silicate glass powder that combines with poly-alkeonic acid liquid. When mixed, it auto-polymerizes, releasing aluminum and calcium ions that form salt bridges and a gel matrix that adheres to mineralized tissues [43,44]. Adhesion to the teeth’s surface is related to ionic forces associated with chelation with calcium [23]. Due to this, Ketac™ is characterized by good adhesion and resistance to fracture [45]. The contraction is initially a slow cross-linked reaction that enables stress relief and maintains a homogeneous and consistent interface between the tooth and the material, leading to reliable adhesion [20]. Despite its high sensibility to manipulation and contraction during polymerization, which sometimes impairs good results, in the present study, this material showed lower levels of infiltration in comparison with Cavit™ and IRM^®^, most likely due to the referred chemical adhesion capacity.

As expected, the resin composite was the material that best prevented microleakage. In this study, an enamel selective etching procedure and a universal dental adhesive containing 10-MDP (Scotchbond™ Universal) were used. The use of functional monomers (such as 10-MDP) in dental adhesives promotes a chemical interaction with both dentin and enamel, improving bond strength and the restoration’s quality and longevity [46,47,48]. The reliable characteristic adhesion of a composite restoration to the tooth structure justified the results. It showed that although temporary materials achieved reasonable results, none of them could present low microleakage results as the ones of definitive restorative materials. This supports the recommendation to perform crown restorations with composites around the access cavity, if necessary, to reduce fluid infiltration at minimum during endodontic treatment [3]. 

As previously stated, published literature on the topic of temporary restorations presents discrepant results. For instance, several studies reported Cavit™ presenting lower infiltration values than IRM^®^ and Ketac™ [15,19,20,40]. Different methodologies may underlie the discrepancies reported in the different studies [14,16,19,49]. It can be explained by the use of different evaluation methods and times and different experimental set-ups to simulate oral conditions, namely thermocycling or/and cyclic loading, the number of cycles in thermocycling, and the materials tested [14,16,19,49].

The infiltration evaluation can be performed using several methodologies, such as methylene blue dye, radioactive isotopes, bacteria, or fluid filtration. However, the use of dyes, mainly methylene blue, is the most frequently used because it is a sensitive indicator of infiltration as it has a small molecule size, similar to the size of microorganisms [14,19]. It has the advantage of not being absorbed by the hydroxyapatite crystal of dentin. Nevertheless, its accuracy depends on how much air is entrapped in the restoration [22]. In addition, this technique has the disadvantage of sectioning the tooth to assess the amount of microleakage, implying a meticulous standardization of the sectioned portions [26]. Bacterial studies are also widely used. However, several different microorganisms are responsible for pulpal infection, and with this method, only a few are evaluated [20]. In the present study, the microleakage was assessed using the infiltration of radioisotope ^99m^TcNaO_4_, a methodology that allows the monitorization and quantification of microleakage at different time points [26,27]. Technetium is a radionuclide with a smaller molecular size comparable to that of the microorganisms present in saliva. It presents selectivity, traveling through the tooth by capillarity and depositing in the “free” areas [26]. It has the advantage over the use of dyes of not requiring the sectioning of the sample, thus allowing the evaluation of the same sample at different time points. This is possible because ^99m^TcNaO_4_ will decay to ^99^TcNaO_4_ which is a stable molecule. In this transition, only energy is dissipated and the molecule does not change. In addition, it presents higher sensitivity compared with the other methods, considering the very small concentrations needed, thus providing more accurate quantitative measures [50]. In fact, the sensitivity is so high that it is only necessary for one atom to be infiltrated for it be detected using a gamma camera. Its main disadvantage is the necessity of specific equipment and radiopharmaceutical availability, which limits its use. Importantly, the results obtained for the positive and negative groups validate the experimental model.

Although the experimental set-up tried to mimic clinical conditions, not all oral variables were reproduced, such as masticatory and occlusal load, which is a limitation. Other analyses, such as push-out tests, could also provide a more comprehensive evaluation. In addition, although the oral cavity has saliva permanently, the restorations could not be submerged entirely but instead were kept in a moist environment, which differs from the protocol used. Other evaluation times could allow more relevant information to be obtained—for instance, shorter times such as 24 h and 7 days. In addition, the increase in thermocycling cycles could better mimic situations where the temporary restorations stay longer in the oral cavity. Finally, in further studies, the use of micro-computed tomography, with superior resolution, will allow a tri-dimensional image of gaps at the tooth/restorative material interface to identify the areas where the restoration/tooth interface fails [51].

Nevertheless, the results obtained in this study are significant and reinforce that temporary restorations should only be used in specific clinical situations and for a short time since their efficacy to prevent microleakage is significantly lower than that of definitive materials. After completing the endodontic treatment, a definitive restoration should be placed as soon as possible.

## 5. Conclusions

Considering the limitations of this ex vivo study, it can be concluded that the three temporary materials present microleakage at 2 and 4 weeks. The experimental hypothesis was rejected since the temporary materials presented different microleakage. Ketac™ Silver presented the lowest infiltration at 2 and 4 weeks, whereas Cavit™ presented a higher infiltration at 2 weeks, diminishing sharply at 4 weeks. At this time, IRM^®^ and Cavit™ prsented similar results. In addition, the second experimental hypothesis was rejected because the definitive material, resin composite, presented significantly lower microleakage than all tested temporary materials.

## Figures and Tables

**Table 1:** Study groups.

Group	Material	Manufacturer	Composition *	Lot Number
1	No material placement	-	-	-
2	Intact teeth	-	-	-
3	IRM^®^	Dentsply Sirona Inc. Milford, DE, USA	powder: zinc oxide, poly-methyl methacrylate (PMMA) powder, pigment liquid: eugenol, acetic acid	powder: 1910001036 liquid: 2001000680
4	Ketac Silver™	3M ESPE, Seefeld, Germany	powder: silver, oxide glass chemicals (non-fibrous), titanium dioxide, copper liquid: water, copolymer of acrylic acid—maleic acid, tartaric acid	7964610
5	Cavit™	3M ESPE, Seefeld, Germany	zinc oxide; sulfuric acid, calcium salt, hydrate; ethylene bis(oxyethylene) diacetate; zinc sulfate; poly(vinyl acetate)	7121289
6	Filtek Supreme™ (+Scotchbond™ Universal)	3M ESPE, Seefeld, Germany	bis-GMA, UDMA, TEGDMA, and bis-EMA resins (MDP phosphate monomer, dimethacrylate resins, HEMA, Vitebond^TM^ copolymer, filler, ethanol, water, initiators, silane)	NC45009 (7676507)

* Information provided by the manufacturer.

**Table 2:** Counts per minute of control and experimental groups after 2 and 4 weeks. Data are presented as mean ± standard deviation. When comparing 4 weeks vs. 2 weeks within each group, ** means *p* < 0.01, and *** means *p* < 0.001.

	G1— Positive Control	G2— Negative Control	G3— IRM^®^	G4— Ketac™ Silver	G5— Cavit™	G6— Filtek Supreme™
2 weeks	20,181.0 ± 2858.9	697.8 ± 222.0	6871.8 ± 1360.2	5615.4 ± 912.3	12,952.9 ± 1199.6	3620.9 ± 702.7
4 weeks	20,769.1 ± 3865.5	669.3 ± 143.3	7561.1 ± 677.2	4236.9 ± 755.6	6807.9 ± 660.2	2927.6 ± 735.1
*p*-value 4 vs. 2 weeks	0.575	0.845	0.114	0.002 **	<0.001 ***	0.059

## Data Availability

Data are contained within the article.
